# The ability of health promoters to deliver group diabetes education in South African primary care

**DOI:** 10.4102/phcfm.v5i1.484

**Published:** 2013-04-23

**Authors:** Anna S. Botes, Buyelwa Majikela-Dlangamandla, Robert Mash

**Affiliations:** 1 Division of Family Medicine and Primary Care, Stellenbosch University, South Africa; 2 Division of Diabetic Medicine and Endocrinology, University of Cape Town, South Africa

## Abstract

**Background:**

Diabetes makes a significant contribution to the burden of disease in South Africa. This study assesses a group diabetes education programme using motivational interviewing in public sector health centres serving low socio-economic communities in Cape Town. The programme was delivered by mid-level health promotion officers (HPOs).

**Objectives:**

The aim of the study was to explore the experience of the HPOs and to observe their fidelity to the educational programme.

**Methods:**

Three focus group interviews were held with the 14 HPOs who delivered the educational programme in 17 health centres. Thirty-three sessions were observed directly and the audio tapes were analysed using the motivational interviewing (MI) integrity code.

**Results:**

The HPOs felt confident in their ability to deliver group education after receiving the training. They reported a significant shift in their communication style and skills. They felt the new approach was feasible and better than before. The resource material was found to be relevant, understandable and useful. The HPOs struggled with poor patient attendance and a lack of suitable space at the facilities. They delivered the majority of the content and achieved beginning-level proficiency in the MI guiding style of communication and the use of open questions. The HPOs did not demonstrate proficiency in active listening and continued to offer some unsolicited advice.

**Conclusion:**

The HPOs demonstrated their potential to deliver group diabetes education despite issues that should be addressed in future training and the district health services. The findings will help with the interpretation of results from a randomised controlled trial evaluating the effectiveness of the education.

## Abstrait

**La capacité des promoteurs de la santé pour offrir une éducation au diabète groupe dans le sud de soins primaires africaine**

**Présentation:**

Le diabète contribue dans une large mesure à la charge de morbidité en Afrique du Sud. Cette étude évalue un programme d’éducation de groupe sur le diabète en utilisant la méthode de l'entretien motivationnel dans des centres de santé du secteur public desservant les communautés socio-économiques désavantagées du Cap. Le programme a été dispensé par des agents de promotion de la santé (APS) de niveau intermédiaire.

**Objectifs:**

L'objectif de l’étude était d’étudier l'expérience des APS et d'observer la mesure dans laquelle ils restaient fidèles au programme d’éducation.

**Méthode:**

Trois sessions de groupe de discussion ont été organisées avec les 14 APS dispensant le programme d’éducation dans 17 centres de santé. Trente-trois sessions ont directement été observées et les bandes audio ont été analysées en utilisant le code d'intégrité relatif à l'entretien motivationnel (EM).

**Résultats:**

Les APS avaient confiance en leur capacité à prodiguer un enseignement à des groupes après avoir suivi la formation. Ils ont observé un changement significatif dans le style et leurs compétences de communication. Ils avaient le sentiment que la nouvelle approche était exploitable et meilleure que la précédente. Les ressources fournies étaient pertinentes, compréhensibles et utiles. Les APS trouvaient que la faible participation des patients et l'absence d'espace adéquat dans les locaux leur rendaient la tâche plus difficile. La majeure partie du contenu était communiquée et ils ont pu atteindre le niveau de débutant en style de communication de l'EM et dans l'utilisation de questions ouvertes. Les APS ne disposaient pas encore d'excellentes compétences d’écoute active et continuaient à fournir des conseils non sollicités.

**Conclusion:**

Les APS ont démontré qu'ils étaient capables de prodiguer un enseignement sur le diabète à des groupes, en dépit de problèmes qui devront être traités au cours de futures formations et dans les services de santé de quartier. Les conclusions contribueront à l'interprétation des résultats obtenus à partir d'un essai contrôlé et aléatoire évaluant l'efficacité de l'enseignement.

## Introduction

In 2010 the International Diabetes Federation reported a prevalence of 4.5% for diabetes amongst South Africans between the ages of 20 and 79 years, with an expected increase to 5.6% by 2030.^1^ However, rates as high as 33% have been reported in some communities in Cape Town.^2^

Diabetes makes a significant contribution to the burden of disease^3^ and is one of the commonest conditions seen in ambulatory primary care.^4^ The complications of diabetes, such as blindness, amputation, and cardiovascular and kidney disease, lead to significant morbidity and mortality.^3^ It is estimated that in the age group 20 years - 79 years, 6% of deaths in Africa were attributable to diabetes in 2010.^1^

The Western Cape is still struggling to improve the quality of care for diabetes. Despite improvements over the last three years, the most recent audit carried out in the Western Cape showed that only 48% of diabetics had an HbA1c test in the previous year and only 35% were controlled (HbA1C < 7%).^5^ Another concerning factor in the audit was that the percentage of facilities providing group education decreased from 90% to 78%.^5^ Health centres that gave didactic talks to groups of mixed patients in the waiting room are likely to have recorded this as group education and so the effectiveness of this education is probably low. Recently it was found that when primary care providers promote lifestyle modification, they often do not have sufficient knowledge to provide practical advice to patients.^6^

Over the last few years several interventions on different aspects of diabetes care have been implemented in the Cape Town Metropolitan District Health Services to try and improve the quality of care. An integrated chronic disease audit and quality improvement cycle was implemented.^5^ The use of a fundal camera for retinal screening in primary care was initiated at some primary care facilities.^7^ An appreciative inquiry process between health workers responsible for diabetic care focused on improving the annual review of the diabetic patient.^8^

However, these initiatives did not focus on preventative measures, like patient education or the empowerment of patients to look after their own diabetes. In a paper about innovative care for chronic conditions published by the World Health Organization in 2002, the following statement was made:^9^

Patients and their families are the most undervalued assets in the health care system. Their potential to affect outcomes is undeniable and their capabilities should be leveraged fully in any model designed to improve care for chronic conditions. They need motivation to change and maintain daily health behaviours, adhere to long-term therapies, and self-manage their conditions.

Different strategies are used to educate patients. A strategy recommended in the new Western Cape Chronic Disease Policy^10^ is to set up community-based support groups via local non-government organisations - with input from the Community Health Centres. The implementation of support groups was seen as a way to improve adherence and increase self-management amongst patients.

Another strategy, suggested by the appreciative inquiry process, was a facility-based diabetes group education programme delivered by health promotion officers (HPOs). The type of information needed and the way in which it should be structured was also discussed during the inquiry process.^8^ The suggestions from the inquiry formed the foundation on which the researchers developed a new education programme. The HPOs are from the community which they serve and they share the same language and culture. They are required to have a secondary school education of at least Grade 10 and to be competent in reading and writing in English. Once employed, the HPOs receive additional in-service training on the knowledge and skills required to deliver health education messages and to promote health. Although the HPOs have experience in health education, their communication style has been one of lecturing, telling, advising or directing patients in what they should do.

Group diabetes education has been proven to be effective in improving diabetes knowledge, reducing the need for diabetes medication, improving HbA1c, increasing weight loss and lowering blood pressure.^12^ However, there are mixed results in the few studies that utilised mid-level health workers^13^ and the literature on group motivational interviewing (MI) is very limited.^14^ In the Cochrane review on group diabetes education, the 12 studies included in the review made use of doctors, sisters and dieticians.12 Groups may not be as effective as individual sessions, but according to Wagner and Ingersoll groups have the following advantages:^14^

- They create a support system for patients.
- They reinforce the notion that the patient is not alone in their experience.
- They may be cost-effective in that fewer counsellors are needed and more patients can be counselled in the time available.

The aim of the study is to explore the experience of the HPOs with regard to the training course and in the facilitation of the group education sessions, and to observe their ability to educate using motivational interviewing skills.

### Contribution to the field

The results of this study will help improve the quality of care for diabetes through preventative measures, like patient education by health promoters that empowers of patients to look after their own diabetes.

## Ethical considerations

Ethical approval for the study was obtained from the Health Research Ethics Committee of Stellenbosch University (N09/10/260).

## Methods

### Study design

Before describing the study design for the work presented, a brief outline of the larger trial to which this study contributes, is given.

#### Pragmatic cluster randomised controlled trial

This study is part of a larger pragmatic cluster randomised controlled trial, which evaluated the effectiveness of the group diabetes education programme delivered by HPOs, using a guiding (motivational interviewing) style in community health centres in Cape Town, South Africa. The study proposal is described elsewhere.^16^ In the larger study, 34 health centres were randomly assigned to either the control or intervention group. Overall, 1570 patients (720 in the intervention group and 850 in the control group) were recruited. Self-efficacy, locus of control, self-care activities, HbA1c, weight, waist circumference, blood pressure, total cholesterol and quality of life were measured at the baseline and also 12 months later.

#### Evaluation of HPOs

This study involved a qualitative exploration by means of focus group interviews (FGI) of the perspectives of the HPOs on their training, as well as the group education sessions. In addition, selected education sessions were observed and audio tapes were analysed to determine the HPOs fidelity to the planned programme, as well as the guiding style.

### Overview of the educational sessions

Patients received a structured education programme of four sessions over a period of one year. These were conducted by the HPOs with groups of 10-15 diabetic patients at a time. The four sessions consisted of the following:

- Session 1: Understanding diabetes.
- Session 2: Understanding the medication.
- Session 3: Living a healthy lifestyle.
- Session 4: Preventing complications.

Group educational material was developed to assist with discussions and group activities. For example, a flipchart assisted with the understanding of diabetes and dietary issues, food cards demonstrated typical local food choices and true or false question cards explored typical local beliefs about diabetes. Education material on foot care, smoking, alcohol use and stress was also available for the patients to take home.

The guiding communication style includes the following characteristics of motivational interviewing:^17^

- Collaboration: both the HPOs and patients should contribute equally to the group discussion.
- Empathy: the HPOs should demonstrate active listening skills and their understanding of the patient's perspective, particularly through the use of summaries.
- Support for autonomy: the HPOs should promote a sense of choice and control over the patients’ ability to change their behaviour.
- Evocation: the HPOs should evoke ‘change talk’ and possible solutions from the group members.
- Direction: the HPOs should manage the time and keep aligned with the intended content and purpose of the sessions.

The sessions were structured around the elicit-provide-elicit strategy for exchanging information.^17^ This strategy involves the following three steps:

- Elicit either the group's prior knowledge or what they are most interested in learning about with regard to a specific topic.
- Provide the group with information in a neutral way that builds on what they already know or that addresses what they are most interested in.
- Elicit how group members will make sense of or apply this information personally.

In addition, the HPOs were trained in the following specific communication skills:

- The use of more open than closed questions.
- The use of summaries and simple reflections.
- The use of affirmations.

### Overview of the training

An initial 4-day workshop was held before they started the educational programme, which focused on the content of the first two sessions and the guiding style of communication. An additional 2-day workshop was held three months later, which focused on the content of the next two sessions and the reinforcement of the guiding style. The training was largely conducted using the same group education skills and strategies so that there was congruence between the approach in the training sessions and the approach to diabetes education. The training sessions were led by R.M. and B.M. with assistance from other experts in behaviour change counselling and diabetes.

### Setting

The study was conducted amongst uninsured patients attending public sector health centres in the metropolitan area of Cape Town. Patients came from low socio-economic backgrounds and spoke either Afrikaans, Xhosa or English. Health centres usually organise a specific day to see diabetic patients in a ‘club’. These ‘clubs’ were originally introduced to deal with the large numbers of diabetic patients and to remove them from the general queue. Patients were evaluated by a nurse and, if necessary, consulted by either a clinical nurse practitioner or a doctor. The group education was offered on the day that patients usually attended the ‘club’. Their usual care included ad hoc education as part of the consultations with the nurse practitioner or doctor, as well as educational talks given to the patients in the waiting room.

### Selection of participants

All 14 HPOs that delivered the group education programme were invited to participate in the FGIs. Some HPOs delivered the education programme in more than one health centre.

Thirty-six sessions out of a total of 68 were selected for observation using a systematic sampling framework that ensured each health centre and each session was sampled equally. For each session selected, the group of patients (A, B, C or D) were then randomly chosen using computer-generated random numbers.

### Procedure

A.B., who did not participate in the training, conducted three FGIs: at the end of the initial training workshop, mid-intervention at the end of the second training workshop and once the education programme was completed. The first FGI explored the perspective of the HPOs on the proposed diabetes programme, the structure of the training workshop, the facilitation and training style of the tutors, any changes made by the HPOs as a result of the training, as well as logistical or organisational issues with regard to the training. The second and third interviews focused on the design of the educational sessions, the resource materials provided, the diabetes content, the communication or facilitation skills, organisational issues and the effect of the education on patients. The interviews were audio taped and transcribed verbatim.

B.M. observed and taped the selected sessions. During the sessions she observed how much of the intended content was actually covered using a structured data collection tool and she kept field notes on issues related to the group facilitation, as well as any other relevant observations.

### Analysis

The transcripts of the FGIs were analysed using theframework method.^18^ The interpretation of the data focused on mapping the range and nature of the phenomenon in terms of the HPOs experience of the education and it looked for explanations for their viewpoints. A summary of the first two interviews was presented to the HPOs at the last interview to validate if the researcher had captured and interpreted their views correctly.

The audio tapes of the actual educational sessions were analysed using the Motivational Interviewing Integrity code version 3.1.1 (MITI).^19^ This is a validated tool for assessing MI with a high reliability on all the elements (Cronbach's alpha > 0.8) and proven sensitivity for detecting changes in counsellor behaviour that are consistent with MI (*p* < 0.05).^19^ The first section made a global rating of the five key characteristics of MI from the whole tape using a Likert scale from 1 to 5, with definitions for each score. A mean global rating could then be calculated. The second section counted specific HPO behaviours throughout the audio tape: open (OQ) or closed questions (CQ), simple reflections or summaries (R) and MI-adherent (MiA) or nonadherent (MiNa) behaviour. MI-adherent behaviours were defined as asking permission, affirmations, emphasising control or offering support. MI-nonadherent behaviour were defined as unsolicited advice and directing or confronting patients. These behaviour counts were then used to calculate summary scores:

- Reflection: Question Ratio (R:Q) = Total reflections/(CQ + OQ).
- Percent Open Questions (% OC) = OQ / (OQ + CQ).
- % MI Adherent (% MiA) = MiA / (MiA + MiNa).

The use of complex reflections was not recorded as these were not part of the training. B.M. was trained in the use of the MITI by R.M. who had previous training and experience with the tool. Inter-rater reliability was addressed by independently coding sessions with feedback of results until the same results were obtained consistently.

## Results

A summary of the HPO demographics are given in Table 1. The majority of HPOs were middle-aged coloured or black women, speaking Afrikaans or Xhosa, with no higher education and an average of 16 years of experience.

**TABLE 1:** Profile of the study participants.

No.	Age (years)	Race	Sex	Educational level	Years of service	Home language
1	49	Coloured	Female	Matric	7	Afrikaans
2	35	African	Female	Matric	4	Xhosa
3	50	Coloured	Female	Degree	23	English
4	58	Coloured	Female	Matric	30	Afrikaans
5	38	African	Male	Matric	4	Xhosa
6	61	Coloured	Female	Matric	20	Afrikaans
7	59	African	Female	Matric	17	Xhosa
8	59	African	Female	Matric	24	Xhosa
9	30	African	Female	Matric	1	Xhosa
10	34	Coloured	Male	Matric	14	Afrikaans
11	49	Coloured	Female	Matric	24	English
12	51	African	Female	Higher Diploma	24	Xhosa
13	50	African	Female	Matric	17	Xhosa
14	48	Coloured	Female	Matric	15	Afrikaans

### The HPO's perspective

All 14 HPOs attended the first interview, 10 the second interview and 8 the final interview. The following themes were derived from the FGIs. The first theme gives feedback on the training course and is followed by feedback on the three main components of the intervention (the communication style, the design of the sessions and the resource material). This is followed by insights into why the programme may have been less successful due to organisational issues and poor attendance, and finally comments on how patients may have changed.

#### Feedback on the training

The HPOs noted that the training process mirrored the desired educational process in the sense that it involved the HPOs in a group process of learning and behavioural change and utilised the same communication skills and strategies. This way of training built their confidence and simulated the situations they would have to face when delivering the sessions in the CHCs. They felt that the training improved the skills they had before:

‘For me also the group participation sort of created the reality of what we are going to be met with when we get there. So it really puts you in that position of preparedness, to be aware of what you are going to go through.’ (FGI 1)

At the end of their training, the HPOs felt confident that they would be able to deliver the four sessions in the CHCs and that their clients would benefit from the proposed diabetes programme:

‘Nothing will prevent me to do this.’ (FGI 1)

The HPOs felt that their knowledge of diabetes improved and that this also built their confidence:

‘I think I have learned more about diabetics, especially [*using*] the flip chart because it shows us how diabetes works in your body. At least I have something now that can build my confidence more and more.’ (FGI 1)

The HPOs had no negative comments regarding the logistical and organisational aspects of the training. They felt that the trainers helped them to understand and comprehend the MI-process:

‘Everything they [*the trainers*] were doing [*related*] to the material, so it was so good. They [*the trainers*] also managed to make us participate in the group session.’ (FGI 1)

#### Changes in communication style and skills

It was evident after the training that most of the HPOs understood the MI-style of interviewing in groups. They felt that there was a shift away from closed-ended questions towards open-ended questions that would encourage elaboration and participation:

‘… making use of more open-ended questions to get more feedback … and interaction from [*the clients*].’ (FGI 1)

The HPOs understood the concept of exchanging rather than transferring information using the strategy of elicit-provide-elicit:

‘… be able to elicit a lot of information from patients and then also be able to correct or provide or even affirm the information that was shared.’ (FGI 1)

The HPOs understood that listening was an important communication skill within MI:

‘I can provide information and then listen some more and then do a summary.’ (FGI 1)

The HPOs conceptualised that they should move away from educating the patients in a teaching or didactic fashion and move towards a style that was more collaborative, facilitatory or guiding:

‘Yes, I am going to change! I am going to be a facilitator now. I am going to ask the clients what do they think about the topic that I am going to talk about and when they give me information, I will just add more and ask them how are they going to change or how are they going to implement that into their lifestyles.’ (FGI 1)

The HPOs felt that the new style improved communication with patients:

‘It could be a good programme because the approach that we use is [very] different and the people understand it much better and we get a lot of input from them. Much more than we used to get when we used the old way of conveying our messages to the people.’ (FGI 3)

However, one HPO felt that the new style of communicating can be time-consuming because some patients want to tell their whole story to the group. Other HPOs felt that it is sometimes difficult to control the talkative patients amongst the less talkative ones:

‘Especially if it is the first person doing the talking - he doesn't want to come across like being rude or what so you have to sort of let them talk.’ (FGI 2)

The HPOs found that it was easier to implement the new communication style than they anticipated:

‘I also never knew that it would be so easy to adapt to the new style of getting your message across. I think it was a very good course.’ (FGI 2)

The patients noticed the new approach and encouraged the HPOs to continue with this:

‘They [*the patients*] asked us “why didn't you do it like this before, we have been here in this institution for a long time, but you didn't do it?”’ (FGI 2)

#### Design of the educational sessions

An overwhelming majority of the HPOs felt that the programme was needed in their setting and that it should continue in the future - even expand to other conditions as well. They felt that an extra topic on sexuality in diabetes should be added in future and that the diabetic patient's partner or a family member should also attend some of the sessions:

‘This is a great programme. I think it should continue and I think it should continue with the other chronic diseases as well.’ (FGI 3)

#### The resource materials

The resource material was regarded as a valuable component of the programme. The HPOs felt that they worked well to illustrate the information. The HPOs used the resource material to restructure the patients’ menus and to show them how big a portion size is. They used the flip charts to explain the pathological process of diabetes:

‘What helped more now is the actual material because now they can look at it. Before they had to visualize it.’ (FGI 3)

#### Organisational issues

Several HPOs had a problem finding a suitable area at their facilities where they could host a group session. The facilities had very few areas suitable for group interaction, especially in the mornings when the health centres were full and space was used for other purposes. Many areas were less than ideal in terms of noise and other disturbances. A few of them made plans to use other facilities in the community, whilst others made special arrangements at their health centres:

‘I also have a problem at my facility about accommodation. I started by going into the community because in our area there is a library and there is a rank office and there is a youth centre. So I went out and tried Plan B because my facility could not accommodate me with a group.’ (FGI 2)

The HPOs felt that it was important to discuss what they were doing with the facility manager and to get the rest of the staff on board. This helped with organisational issues such as the timely dispensing of patients’ medication by the pharmacist on the same day that the group session takes place. Some HPOs had trouble with the pharmacists, who felt that the patients attending the sessions should not be treated differently than other patients. However, one pharmacist provided an incentive so that patients who attended the education could be fast-tracked for the rest of the year:

‘But you know what, you can arrange if somebody comes to the group, and their medication is for the Friday or whatever, I would go to the Facility Manager, not doing things on my own because then you get into trouble. I would give the cards to her and she would arrange that.’ (FGI 2)

#### Poor attendance and its consequences

All the HPOs had problems with poor attendance and people coming on the wrong dates for their group sessions or attending the sessions out of sequence. This caused the HPOs to restructure their sessions. The HPOs had to be prepared for any one of the four sessions on a particular day, because they did not know which sessions the patients previously attended:

‘This is my bag, everything is in here. So if they already had session one or most of the people must have session three, everything is in this bag. We actually said it is a good idea because now you come and you must bring session two's things, but then you see that most of them have session three here. So everything was in here so you just pull out whatever session you must do. That is how I coped with it.’ (FGI 3)

The HPOs gave their feedback on why they thought the patient attendance was poor:

- Patients were not contacted to inform them of when they were expected to come for the next session.
- Patients were working and could not get time off from work to attend the sessions.
- Patients shared cell phones with family members and then never received the SMS, or the message was delayed by the network and delivered too late.

The HPOs made a number of suggestions to improve the educational programme, which were mainly aimed at increasing attendance:

- The HPOs felt that there should be a standardised attendance certificate that they could hand out to patients who were working.
- The HPOs suggested that the patients should rather be phoned than sent a text message on their cell phones.
- The HPOs wanted to have a glucose monitoring machine available for each group where they could test the patients as a motivation for the patients to lower their blood glucose and attend the sessions.

#### Perceived changes in the patients

The HPOs reflected on a number of changes that they saw in the patients as a result of the educational programme. The HPOs perceived that the sessions improved patient's confidence and gave them a sense of control over issues such as their diet and medication, despite their circumstances:

‘I am 58 years of age. I am a diabetic for about 10 years and since I attended the sessions with [*the HPO*] it gets more understandable for me. For my first few years I did not understand anything about the diabetic, but since I have attended the sessions with [*my* HPO] it gets more interesting and I feel myself. I just want to thank [*my HPO*] for guiding me and giving me more information about diabetic. I know how to eat and how to use my medication.’ (A letter one of the HPOs received from a grateful patient)

The sessions helped the patients to understand their medication, leading to increased overall adherence to treatment. Weight loss was reported amongst several patients with the commitment to a change in their diet and physical activity. One HPO started an exercise programme with the group after the patients recognised that they needed to exercise more. Some reported smoking cessation after the programme was delivered:

‘She [*a patient*] is now taking the medication and she is taking the insulin as prescribed.’ (FGI 3)

‘One of them lost a lot of weight and some of them are still trying and … some of them [*were*] saying that [*they*] eat all the healthy stuff, but it is just that they are overeating.’ (FGI 3)

The HPOs also reported observable improvement in patients’ blood glucose levels:

‘Their sugar levels … really dropped.’ (FGI 3)

#### Observation of the HPOs

Thirty-three sessions were observed as the HPO resigned at one CHC and these sessions were cancelled. On average the HPOs delivered 89.6% of the content for ‘understanding diabetes’, 87.5% for ‘understanding medication’, 78.1% for ‘understanding complications’ and 76.7% for ‘healthy lifestyle’. Table 2 shows their fidelity to MI compared to the thresholds recommended in the MITI tool. The HPOs demonstrated borderline beginning-level proficiency in terms of the overall guiding style and its characteristics as shown by the global ratings. Within the overall global rating, their mean scores were the highest for direction (4.7 SD0.5) and collaboration (3.6 SD0.8), and the lowest for evocation (3.4 SD0.7), supporting autonomy (3.1 SD0.7) and empathy (2.8 SD0.7). The HPOs demonstrated an ability to ask more open than closed questions and to achieve beginning-level proficiency. They did not demonstrate sufficient active listening skills as shown by the low reflection-to-question ratio and continued to offer some unsolicited advice, as well as the use of inappropriate directing as shown by the low MI-adherence percentage.

**TABLE 2:** Fidelity to the guiding style.

Variable	Target for beginning-level proficiency	Target for competency	Results for sessions 1-2		Results for sessions 3-4	
				
			Mean score	s.d.	Mean score	s.d.
Global rating (1–5)	3.5	4	3.5	0.50	3.4	0.44
Percent open questions	50	70	65.4	13.0	64.6	13.9
Reflection to question ratio	1	2	0.1	0.08	0.1	0.09
Percent MI adherent	90	100	59.6	36.5	38.7	33.6

s.d., standard deviation.

The observed sessions confirmed that patient attendance was a problem - group sizes were usually less than 10 people and patients attended the sessions out of sequence, such that the HPOs had to adapt to what people had previously learned. The lack of a suitable area to host the sessions was noted as a major problem - the HPOs either had no suitable space in the facility or the space was too small, noisy or interrupted. The HPOs made good use of the resource material and the summary sheets of each session that prompted them on what to do. Sessions lasted between 20 and 60 minutes. Language was not usually an issue as the HPOs conducted the session in the patient's first language. Occasionally the group had mixed preferences so that two languages had to be used, which took more time. In a few instances the HPO did not speak Xhosa, which made communication with some participants difficult.

## Discussion

### Key findings

From the perspective of the HPOs this was a viable and effective educational programme. It was evident that the HPOs felt that the training process in group MI was effective and that it equipped them with the necessary confidence and skills to deliver the sessions.

The overall design of the sessions was supported and the majority of the intended content was covered. Additional topics and the involvement of family members were suggestions made. The HPOs perceived the resource material used in the study as relevant and understandable and it was used widely. They experienced the implementation of the group MI-style as easier than expected and the patients attending the sessions reported positive feedback on the change in style. The HPOs clearly made a significant shift from their previous approach to health education and became more collaborative. Nevertheless, they did not achieve beginning-level proficiency on all the MI measures and continued to offer some unsolicited advice. They also struggled to offer summaries and simple reflections.

Feedback was dominated by frustration with poor patient attendance, discussing the reasons for this and suggestions to overcome this problem. Outside of a research study, a more pragmatic approach to attendance could be adopted on an ongoing basis that would allow patients to attend more spontaneously and at times that are convenient for them.

Problems at the CHCs were mostly based on space issues - this needs to be addressed in future. Many CHCs appear to have prioritised areas for other objectives, such as HIV and AIDS and trauma care, but non-communicable diseases are less of a priority when allocating space. The HPOs developed innovative ways of solving these problems, although they needed to obtain support from other more powerful stakeholders at the facility.

Previous studies on group diabetes education used doctors, nurses or dieticians to facilitate groups,^12^ whilst this and other studies suggest that HPOs or mid-level workers may be a viable alternative in lower resource settings.^6^ The finding that mid-level workers particularly struggle to learn active listening skills was also showed in a study on HIV counsellors and MI.^21^ Future training should spend more time on the use of summaries and simple reflections.

The manager of the HPOs requested that the other HPOs also receive this training as she noticed their improved confidence in diabetes education.

### Strengths and limitations of the study

A.B. analysed and interpreted the interview data with supervision from R.M. and this may have increased the probability of personal assumptions, values and beliefs influencing the outcome. However, A.B. was not part of the training sessions or the larger study and this should have elicited honest responses from the HPOs. Respondent validation after the first two sessions confirmed that the initial interpretation of the data was correct. English was used for the FGIs and, although the HPOs were fluent in English, it was their second language.

The MITI tool was originally validated for assessment of individual MI counselling and its use here was extended to MI in a group setting. Nevertheless, the contents of the tool remained congruent with the objectives of the training and what was expected from the HPOs. The only adaptation was the removal of complex reflections.

### Implications and recommendations

The results provide useful feedback on the fidelity of the HPOs to the planned educational programme which will assist with the interpretation of the randomised controlled trial. A number of issues have been identified that can be incorporated into future training for HPOs. The issue of space in primary care facilities for the education of patients with non-communicable chronic diseases needs to be addressed by the district health services.

## Conclusion

The HPOs perceived the approach to training to be successful and felt confident in their ability to deliver the diabetes education programme. The most useful components of the programme were the new communication style and skills and the resource material that improved the patients’ ability to understand their disease and to plan self-care activities. Poor patient attendance and a lack of available space in the facilities were problems in the programme's effective delivery. The HPOs delivered the majority of the content and showed beginning-level proficiency in MI in terms of their overall guiding style and the use of open-ended questions. The HPOs struggled with active listening and continued to offer some unsolicited advice.
